# Changes in personality traits in patients with Alzheimer's Disease

**DOI:** 10.1590/1980-5764-DN-2021-0029

**Published:** 2022-04-29

**Authors:** Kaoue Fonseca Lopes, Valéria Santoro Bahia, Jean Carlos Natividade, Rafael Valdece Sousa Bastos, Wanderley Akira Shiguti, Kátia Estevão Rodrigues da Silva, Wânia Cristina de Souza

**Affiliations:** 1 Universidade de Brasília, Instituto de Psicologia, Departamento de Processos Psicológicos Básicos, Brasília DF, Brazil.; 2 Clínica de Neurologia Neurob, Brasília DF, Brazil.; 3 Universidade Cidade de São Paulo, Departamento de Medicina Interna, São Paulo SP, Brazil.; 4 Pontifícia Universidade Católica do Rio de Janeiro, Instituto de Psicologia, Departamento de Psicologia Social, Rio de Janeiro RJ, Brazil.; 5 Universidade São Francisco, Instituto de Psicologia, Departamento de Psicologia Social, São Paulo SP, Brazil.; 6 Centro Universitário IESB, Brasília DF, Brazil.

**Keywords:** Alzheimer Disease, Personality Inventory, Mental Status and Dementia Tests, Neuroticism, Extraversion, Psychological, Doença de Alzheimer, Inventário de Personalidade, Testes de Estado Mental e Demência, Neuroticismo, Extroversão Psicológica

## Abstract

Changes in personality traits in patients with Alzheimer's disease (AD) are extremely common throughout the course of the pathology, and these behavioral changes present themselves as challenges in clinical management and as a significant cause of caregivers’ burden.

**Objective:**

Using a personality inventory based on the five-factor model of personality, this study aimed to assesses the change in these factors by comparing the premorbid and current personality of individuals recently diagnosed with AD.

**Methods:**

A total of 30 AD patients were recruited, and their respective family members responded to the personality inventory at home through a hosted site. The patients were also divided into two groups according to the Clinical Dementia Rating (CDR): mild dementia (CDR 1) and moderate dementia (CDR 2).

**Results:**

Among all patients, there was a significant increase in neuroticism factor levels and a significant decrease in the extraversion, conscientiousness, openness, and socialization factors. When comparing the groups, only the extraversion factor showed a difference, with CDR 1 group accusing a higher change in scores. Higher scores in the factor neuroticism in the premorbid personality correlated with the current severity of the disease.

**Conclusions:**

This research draws the attention of family members and health professionals to changes in personality traits or behavior of relatives or patients, because it can reflect an underlying neurodegenerative process.

## RESUMO.

Mudanças em traços de personalidade em pacientes com doença de Alzheimer (DA) são extremamente comuns ao longo do curso da referida patologia, e essas alterações comportamentais apresentam-se como desafios no manejo clínico e como causa significativa de esgotamento dos cuidadores.

**Objetivo:**

Por meio de um inventário de personalidade baseado nos cinco fatores de personalidade, este estudo avalia a mudança nos escores desses fatores comparando a personalidade pré-mórbida e a atual dos indivíduos com DA.

**Métodos:**

O total de 30 pacientes com DA foi recrutado, e seus familiares responderam ao inventário de personalidade. Os pacientes também foram divididos em dois grupos conforme a avaliação clínica da demência: demência leve (CDR1) e demência moderada (CDR2).

**Resultados:**

Em todos os pacientes, houve aumento significativo nos escores do fator neuroticismo e decréscimos significativos nos fatores extroversão, realização, abertura e socialização. Quando feita a comparação entre grupos, apenas o fator extroversão apresentou diferença, com o grupo CDR 1 mostrando maiores mudanças nos escores. Os níveis do fator neuroticismo da personalidade pré-mórbida correlacionaram-se com a gravidade da doença no momento do diagnóstico.

**Conclusões:**

Este estudo procura esclarecer aos familiares e profissionais de saúde que mudanças em traços de personalidade de seus parentes ou pacientes podem refletir processos neurodegenerativos subjacentes.

## INTRODUCTION

Personality traits are generally consistent in adulthood and old age, although small changes in personality occur throughout life ^1^. However, progressive changes are not typical and can mean underlying neurological disease, such as Alzheimer's disease (AD)^2^. Thus, it is common for close relatives to observe significant changes in the personality of individuals who develop dementia^3^.

In the seminal case described by Alois Alzheimer, Auguste D's husband observed significant behavioral disorders, including paranoia, crying, aggression, and other unpredictable behaviors. As described in this first case, changes in behavior and personality remain the most challenging clinical symptoms in the treatment of dementia^4,5^. In this context, clinical research has studied the influence of AD dementia syndrome on changing personality traits^6–8^.

One means of assessing personality change has been through retrospective studies in which an experienced informant, usually the spouse or child, assesses the premorbid and current personality of the person with dementia^9,10^. These reports from informants play a critical role in clinical assessments and are an important source of information to characterize the current state of the patient and the changes that have occurred over time^11,12^. In this sense, to compare the changes that occurred between the premorbid and current personality, the assessment of characteristics based on the five-factor model of personality is widely used (also known as the Big Five)^10^. This model anchors personality to the following factors: neuroticism (the tendency to experience negative emotions such as fear and sadness), extroversion (tendency to be outgoing, social, and energetic), openness (tendency to prefer new and diverse experiences and have intellectual curiosity), socialization (tendency to being cooperative, kind, and confident), and conscientiousness (tendency to be organized, persistent, and careful). Narrower traits, called facets, are part of each of the five dimensions. For example, neuroticism includes facets that reflect anxiety, angry hostility, depression, self-awareness, impulsiveness, and vulnerability^13–15^.

As designed in this research, other studies have investigated the change in each of the five characteristics over the course of AD^15^ and have shown associations between changes in the factors’ scores with cognitive decline. Similar patterns were observed in individuals diagnosed with mild cognitive impairment (MCI) but to a lesser extent^16–18^. Indeed, the personality dimensions or factors shape, throughout an individual's life, contexts of reactions to stress, engagement in physical, cognitive, and social activities, and situations that are related to AD^19^.

Thus, the objective of this study was to advance knowledge regarding the changes in personality factors that occur in individuals diagnosed with AD, reported by close relatives, with the hypothesis of an increase in the levels of the neuroticism factor and a decrease in the levels of openness, extraversion, conscientiousness, and socialization.

## METHODS

### Sample and procedures

A total of 30 individuals with cognitive complaints were attended by a neurologist and diagnosed with dementia due to AD based on Mckhann's criteria^15^ at an outpatient clinic. They were recruited for the study with family consensus. Mini-Mental Status Examination (MMSE)^20^ and Clinical Dementia Rating (CDR) scale^21^ were applied to patients, and also the age and level of education data were collected.

After medical consultation, spouse or children who lived with the patient for at least 15 consecutive years responded to the Personality Factorial Battery (PFB)^10^, a psychometric instrument based on the model of the five major personality factors validated for the Brazilian population with excellent internal consistency and test–retest reliability^22^.

The battery comprises 126 items in a seven-point Likert format for family members to judge how much each statement applies to the participants, and it was responded in patients’ home through a hosted site on Internet. Family members responded to statements about patients’ personalities and characteristics in two times: thinking about 10 years ago when participants were asymptomatic (t1) and thinking about the present when the patients received the diagnostic AD (t2). Patients were also separated into two groups: mild dementia (CDR 1) and moderate dementia (CDR 2).

Patients were excluded from the research if they had CDR 3, had moderate-to-advanced cerebrovascular disease^23^, or were not using psychotropic medications in a stable manner for at least 3 months. Individuals with a positive polymerase chain reaction (PCR) test for COVID-19 in the past 14 days or with flu-like symptoms on the day of the medical appointment were also excluded. In addition, patients who were analphabets or without Internet access were excluded.

The study was approved by the Research Ethics Committee of the Institute of Human Sciences of the Universidade de Brasília.

### Data analysis

The *Statistical Package for the Social Sciences* (SPSS) software, 21st version, was used to analyze database. The means and standard deviations for the personality factors’ scores were calculated in t1 and t2, and paired t-tests for one sample were performed to verify the differences in means, assuming a significance level equal to 5%. Scores for all factors and subfactors were reported in relation to the normative sample of the PFB.

Comparative analyses through multivariate analysis of variance (MANOVA) and two-sample t-test were performed to evaluate the existence of significant differences of the means between group scores.

Linear regression was used to access possible confounders of age and schooling between the groups. Correlations through the groups were conducted by Pearson's coefficient.

## RESULTS

As shown in Table 1, the study included 30 patients with AD, 18 of whom were women. The mean age was 71.9 years (SD=7.4), and the mean MMSE was 22.1 (SD=3.1) and 14.5 (SD=2.5) in the mild dementia group and the moderate dementia group, respectively.

**Table 1:** Clinical Dementia Rating, Mini-Mental Status Examination, gender, and age scores of the patients.

Female			Total		
**Participants**	**Mean**		**Participants**	**Mean**	
	**Age Mean (SD)**	**MMSE Mean (SD)**		**Age Mean (SD)**	**MMSE Mean (SD)**
8	75.9 (5)	20.6 (2)	**16**	70.9 (8.6)	22.1 (3.1)
10	74.1 (5.4)	13.8 (1.8)	**14**	73 (5.8)	14.5 (2.5)
18	74.9 (5.1)	16.8 (3.9)	**30**	71.9 (7.4)	18.5 (4.7)

MMSE: Mini-Mental Status Examination; SD: standard deviation.

Regarding the education of patients, 9.7% had completed higher education, 9.7% had incomplete higher education, 22.6% had completed high school, 12.9% had completed elementary school, and 45.2% had incomplete elementary schooling, and the groups were homogeneous in terms of age [t(30)=-0.29; p=0.78] and level of education [t(30)=1.30; p=0.20].

In the paired t-test analysis of the 30 patients, significant differences were verified between the premorbid state and the current state in all the factors (Table 2). Regarding the subfactors, kindness[t(30)=-5.37; p<0.001], dynamism [t(30)=-8.48; p<0.001], social interactions [t(30)=-6.80; p<0.001], competence [t(30)=-9.53; p<0.001], and endeavor/commitment [t(30)=-4.75; p<0.001] showed higher levels of changes. In contrast, there was no significant difference between the scores of the subfactors trust in people before and after [t(30)=-1.49; p=0.14], arrogance before and after [t(30)=-1.64; p=0.11], vulnerability before and after [t(30)=0.44; p=0.66], and search for novelty before and after [t(30)=-1.70; p=0.090].

**Table 2:** Comparison between premorbid and current personality factors.

Personality facets		10 years ago	Currently	p-value	R	D
		Mean (SD)	Mean (SD)			
Socialization		5.55 (1.02)	4.59 (1.17)	<0.001	0.40	-0.87
Kindness		5.77 (1.44)	4.20 (1.52)	<0.001	-0.47	-1.06
Pro-sociability		5.85 (0.90)	5.25 (1.23)	0.001	-0.27	-0.56
Trust in people		4.90 (1.35)	4.52 (1.48)	0.14*	-0.13	-0.27
Extraversion		4.66 (1.06)	3.21 (0.96)	<0.001	0.58	-1.43
	Communication	4.56 (1.33)	3.50 (1.18)	0.001	-0.39	-0.84
	Dynamism	5.34 (1.35)	2.93 (1.24)	<0.001	-0.68	-1.86
	Social interactions	4.93 (1.42)	2.92 (1.06)	<0.001	-0.63	-1.62
	Arrogance	3.96 (1.29)	3.45 (1.29)	0.11*	-0.19	-0.39
Conscientiousness		5.26 (1.09)	3.03 (1.17)	<0.001	0.70	-1.97
	Competence	5.63 (1.23)	2.72 (1.47)	<0.001	-0.73	-2.15
	Reflection/Prudence	4.63 (1.38)	3.60 (1.17)	<0.001	-0.37	-0.80
	Endeavor/Commitment	5.07 (1.31)	3.16 (1.47)	<0.001	-0.56	-1.37
Neuroticism		3.52 (1.05)	4.39 (1.02)	0.001	0.84	0.39
	Emotional instability	3.65 (1.80)	4.64 (1.62)	0.006	0.28	0.59
	Passivity	3.63 (1.34)	4.78 (1.20)	0.001	0.41	0.90
	Depression	2.85 (1.36)	4.34 (1.55)	<0.001	0.45	1.02
	Vulnerability	3.93 (1.11)	4.02 (1.08)	0.66*	0.04	0.08
Openness		3.69 (0.83)	3.18 (0.70)	0.002	0.31	-0.66
	Openness to new ideas	3.74 (1.15)	3.12 (1.12)	0.006	0.55	-0.26
	Liberalism	3.56 (1.05)	3.17 (0.96)	0.011	0.39	-0.19
	Search for novelty	3.61 (1.03)	3.31 (1.09)	0.09*	0.29	-0.14

* p<0.05;

SD: standard deviation.

In the MANOVA analysis (Table 3), both groups showed significant changes in the five factors across t1 and t2, with an increase in the neuroticism scores and a decrease in the other four factors.

**Table 3:** Comparative analysis between the groups.

Analysis		Neuroticism		Extroversion		Socialization		Conscientiousness		Openness	
		F	p-value	F	p-value	F	p-value	F	p-value	F	p-value
MANOVA											
	Time	12.559	0.001	36.006	0.000	19.416	0.000	74.705	0.000	11.455	0.002
	CDR×time	0.336	0.567	6.217	0.019	0.436	0.515	3.861	0.059	2.368	0.135
Paired comparison between subjects											
	CDR		0.009		0.644		0.378		0.775		0.880

F: statistical value; CDR: Clinical Dementia Rating; MANOVA: multivariate analysis of variance.

**Figure 1:**
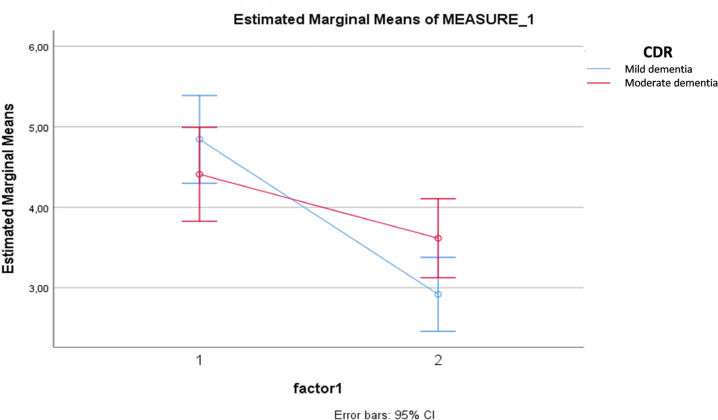
Difference in the extroversion factor scores between the groups.

When comparing the two groups, only the factor extraversion showed a significant change in the scores across t1 and t2 (Figure 1).

**Figure 2:**
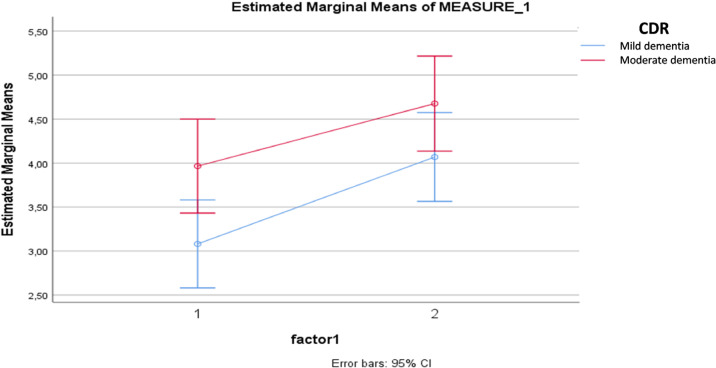
Difference in the neuroticism factor scores between the groups.

When considering just the CDR, higher scores in neuroticism in t1 correlated with more numbers of patients with moderate dementia in t2 (Figure 2).

## DISCUSSION

The present study investigated the influence of AD on the change in personality traits of outpatients with this pathology. The results obtained revealed a significant increase in the scores of neuroticism and a significant decrease in the level factors, such as extraversion, conscientiousness, openness, and socialization, when the factors were compared between the premorbid and current personality^24,25^.

The data found are consistent with most studies similarly outlined in the literature. They have shown remarkable patterns of personality change and huge differences when comparing AD patients with normal aging^17^.

Functional neuroimaging studies correlate personality factors with structural dimensions and degrees of large networks activation. As an example, low levels in conscientiousness correlated with an increase in cerebral white matter lesions and in a reduced volume of the dorsolateral prefrontal cortex^13,18^. In turn, it is important to remember that characteristics of conscientiousness or neuroticism are factors related to circumstances like smoking, physical inactivity, obesity, and depressive symptoms, which are established risk factors for dementia^26,27^.

In this sense, personality traits can also have an impact on the concept of individuals’ cognitive reserve due to poorly adaptive behaviors or activities related to intellectual curiosity, creativity, interest in places, and social ties. This concept links the resilience with individual behaviors of AD pathologies that can be protective or disruptive. In the other way, incipient AD pathology can degenerate important cerebral areas and provide deleterious effects, such as the behavioral and psychological symptoms of dementia. For these reasons, studies on neuroimaging and clinical personality assessment help elucidate this intricate relationship between personality and cerebral structure and function^17,28^.

Maheen et al.^29^, in a meta-analysis with 592 patients, where personality changes were reported retrospectively from the informant perspective, found a replicable evidence for large changes in personality among individuals with AD, particularly decrease in extraversion and conscientiousness and increase in neuroticism. Seeking for responses or even more questions, Terraciano and Sutin^17^ reviewed prospective studies and found elevated scores in neuroticism and decreased scores in conscientiousness as independent risk factors for dementia.

Future research should continue to examine whether different patterns of personality changes across etiologies of dementia and prospective assessments of personality using both self-report and informant report are needed to interpret this mental trajectory built by personality and cognition^30^.

In the context of this study, family members of patients with mild dementia noticed a greater decrease in extroversion compared with family members of patients with moderate AD. This fact may suggest that in mild AD, changes in behaviors related to communication, dynamism, and social interactions are perceived in a more intense way by family members. There was also a correlation between higher levels of premorbid neuroticism, which favors characteristics such as emotional instability and vulnerability, with a greater severity of the disease^17,18^.

Some important limitations in this study include a small sample, data collection based on information from third parties, and the absence of a control group and retrospective design that makes it difficult to establish causalities. For this purpose, prospective studies are needed to be done, including individuals with MCI and other types of dementia. However, in conclusion, it is a research design that brings clinical information regarding personality changes in the course of dementia processes. The early identification of these changes can assist clinicians in choosing tailored interventions to mitigate the psychological distress of their patients together with preventive coping strategies to avoid harmful behaviors.
